# Simulations of a protein fold switch reveal crowding-induced population shifts driven by disordered regions

**DOI:** 10.1038/s42004-023-00995-2

**Published:** 2023-09-09

**Authors:** Saman Bazmi, Bahman Seifi, Stefan Wallin

**Affiliations:** Department of Physics and Physical Oceanography, Memorial University of Newfoundland, St. John’s, NL A1B 3X7 Canada

**Keywords:** Biophysical chemistry, Biophysical chemistry, Computational chemistry, Protein folding

## Abstract

Macromolecular crowding effects on globular proteins, which usually adopt a single stable fold, have been widely studied. However, little is known about crowding effects on fold-switching proteins, which reversibly switch between distinct folds. Here we study the mutationally driven switch between the folds of G_A_ and G_B_, the two 56-amino acid binding domains of protein G, using a structure-based dual-basin model. We show that, in the absence of crowders, the fold populations *P*_A_ and *P*_B_ can be controlled by the strengths of contacts in the two folds, *κ*_A_ and *κ*_B_. A population balance, *P*_A_ ≈ *P*_B_, is obtained for *κ*_B_/*κ*_A_ = 0.92. The resulting model protein is subject to crowding at different packing fractions, *ϕ*_c_. We find that crowding increases the G_B_ population and reduces the G_A_ population, reaching *P*_B_/*P*_A_ ≈ 4 at *ϕ*_c_ = 0.44. We analyze the *ϕ*_c_-dependence of the crowding-induced G_A_-to-G_B_ switch using scaled particle theory, which provides a qualitative, but not quantitative, fit of our data, suggesting effects beyond a spherical description of the folds. We show that the terminal regions of the protein chain, which are intrinsically disordered only in G_A_, play a dominant role in the response of the fold switch to crowding effects.

## Abstract (web summary)

Proteins that are able to switch between different folds correlate their structural and functional changes, however, crowding effects on fold-switching proteins are not fully understood. Here, the authors study the fold switch of the protein G domains G_A_ and G_B_ using a structure-based dual-basin model, revealing a crowding-induced G_A_-to-G_B_ fold switch, and elucidating the role of a disordered range in G_A_.

## Introduction

Most globular proteins rely on a single fold to carry out their function. However, recently proteins have been discovered with an ability to switch between different folds^1–4^, a phenomenon called fold switching. By adopting an alternative structure, these fold-switching proteins (also termed metamorphic^5^ or transformer^6^ proteins) gain the ability to carry out an additional unrelated function. For example, a switch from a helical hairpin to a *β*-barrel transforms the *Escherichia coli* protein RfaH from a transcription factor to a translational activator^7^. Consistent with this view, fold switching is often regulated^8^. A range of cellular signals has been associated with fold switching, such as changes in salt concentration^9^, redox conditions^10^, and oligomerization^11^. Fold switching also underpins evolutionary changes in protein structure^12–14^, in which case fold switching is driven by mutations.

**Fig. 1:**
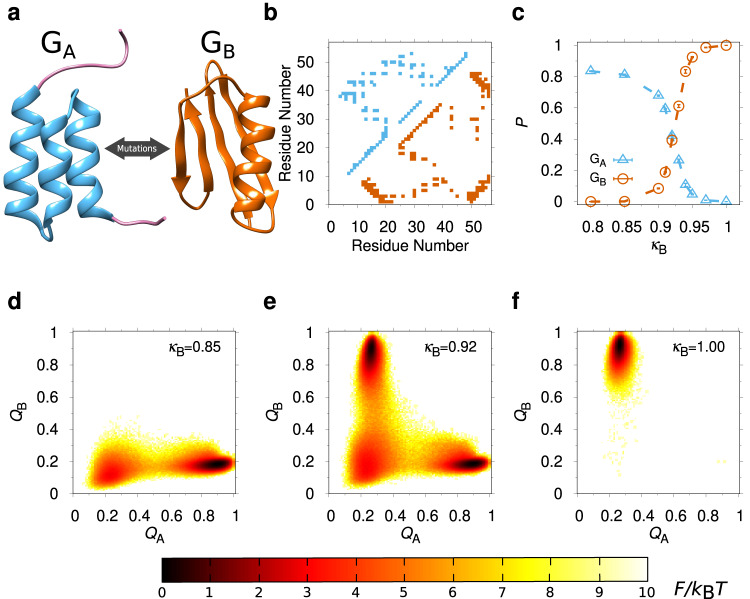
Simulating the G_A_/G_B_ fold-switch system. **a** Representative experimental structures of the G_A_ and G_B_ folds shown in ribbon: G_A_95 (PDB id 2KDL; blue) and G_B_95 (PDB id 2KDM; orange). In G_A_95, residue positions 1-7 and 53-56 are intrinsically disordered (purple). **b** Contact maps of the G_A_95 (above diagonal) and G_B_95 (below diagonal) structures. **c** Population *P* of the G_A_ (triangles) and G_B_ (circles) folds as functions of the G_B_ contacts strength, *κ*_B_. **d**–**f** Free energy surface $F({Q}_{{{{{{{{\rm{A}}}}}}}}},{Q}_{{{{{{{{\rm{B}}}}}}}}})=-{k}_{{{{{{{{\rm{B}}}}}}}}}T\ln P({Q}_{{{{{{{{\rm{A}}}}}}}}},{Q}_{{{{{{{{\rm{B}}}}}}}}})$, where *Q*_A_ and *Q*_B_ are the fractions of G_A_ and G_B_ contacts, respectively, *T* is the temperature, *k*_B_ is the Boltzmann constant, and *P*(*Q*_A_, *Q*_B_) is a probability distribution, obtained at three different values of *κ*_B_. Results in (**c**)–(**f**) are taken at temperature *T*_0_ (in model units, *k*_B_*T*_0_ = 0.88, where *k*_B_ is the Boltzmann constant). Error bars in (**c**) and other figures unless otherwise stated, represent 1*σ* standard error of the mean estimated from independent simulations.

In this work, we investigate the effects of macromolecular crowding on fold switching. To this end, we focus on the binding domains of protein G, G_A_ and G_B_, which form one of the most well-characterized fold switch systems^15^ (see Fig. 1a). It was demonstrated that a set of substitution mutations can be found which drastically increases the sequence identity of G_A_ and G_B_, while still retaining their respective native structures and binding partners^15^. For example, the variants G_A_95 and G_B_95 differ in only 3 amino acid positions. Hence, three additional substitutions (L20A, I30F and L45Y) applied to G_A_95 cause an abrupt switch from the 3*α* fold of G_A_ to the 4*β* + *α* fold of G_B_. Later it was shown that there are multiple ways in which a single substitution can tip the balance from one fold to the other, e.g., L20A applied to the variant G_B_98-T25I^16^. These experiments on G_A_ and G_B_ were, however, carried out in dilute protein solutions and therefore in the absence of any crowding effects.

We carry out our simulations with a coarse-grained structure-based model, which we develop and test on the G_A_/G_B_ fold switch in the absence of crowders (see “Methods”). The structure-based approach involves constructing a potential energy landscape with a single basin of attraction by making native contacts attractive and non-native contacts repulsive. This type of modeling has provided important insights into several aspects of protein folding^17–20^. The natural extension to fold switching is a potential with dual basins of attractions corresponding to the two alternative folds^21–26^. Our dual-basin model for G_A_/G_B_ fold switching permits us to mimic the progression of mutations along a pathway from one fold to the other by tuning the relative interaction strengths of residue-residue contacts in the G_A_ and G_B_ folds (see Fig. 1b). To understand the effect of crowding, we focus on the point along the mutational pathway where the G_A_ and G_B_ folds exhibit roughly equal fold propensities, which we reasoned should be especially susceptible to crowding effects.

## Results

### Mimicking the mutational pathway between the G_A_ and G_B_ folds

We first simulate the G_A_/G_B_ system in the absence of crowders at a fixed temperature, *T*_0_, sufficiently low for low-energy folded conformations to dominate over those in the unfolded state (U). By varying the strength *κ*_B_ of G_B_ contacts, keeping the strength of G_A_ contacts fixed (*κ*_A_ = 1), we can control the relative population of the two folds in our model, as shown in Fig. 1c. While G_B_ is the dominant state at high *κ*_B_ (≳0.97) G_A_ dominates at low *κ*_B_ (≲0.85), where there is also a non-zero population of U. At an intermediate value, *κ*_B_ = κ^*^ = 0.92, the populations of G_A_ (*P*_A_) and G_B_ (*P*_B_) are almost equal, *P*_A_ ≈ *P*_B_ ≈ 0.39–0.42. The drastic population shifts between states G_A_, G_B_, and U, can be seen from the free energy surfaces *F*(*Q*_A_, *Q*_B_), where *Q*_A_ and *Q*_B_ are the fractions of formed G_A_ and G_B_ contacts, respectively, taken at different *κ*_B_ values (see Fig. 1d–f).

**Fig. 2:**
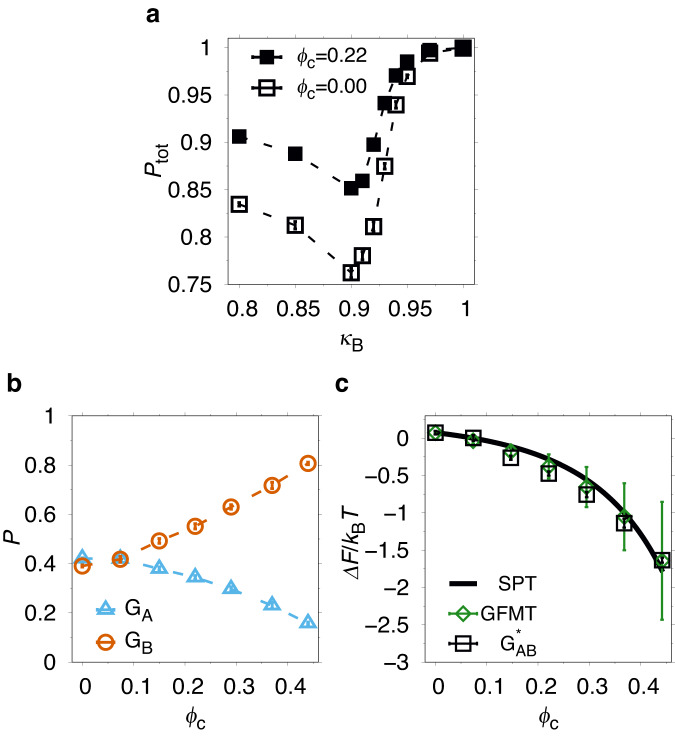
Crowding effects on total and relative fold populations. **a** The total native population *P*_tot_ = *P*_A_ + *P*_B_ as function of the contact strength *κ*_B_ in the absence (open squares) and presence (filled squares) of crowders at packing fraction *ϕ*_c_ = 0.22. **b** G_A_ (*P*_A_; triangles) and G_B_ (*P*_B_; circles) fold populations as functions of *ϕ*_c_ for model protein ${{{{{{{{\rm{G}}}}}}}}}_{{{{{{{{\rm{AB}}}}}}}}}^{* }$. **c** Free energy of fold switching $\Delta {F}_{{{{{{{{\rm{switch}}}}}}}}}=-{k}_{{{{{{{{\rm{B}}}}}}}}}T\ln {P}_{{{{{{{{\rm{B}}}}}}}}}/{P}_{{{{{{{{\rm{A}}}}}}}}}$ (squares) as function of *ϕ*_c_, fitted to Eq. (4) with *δ* as a single free parameter (solid curve). Green rhombuses are average Δ*F*_switch_ values calculated using GFMT (Eq. (5)) for a representative set of G_A_ and G_B_ conformations taken from simulations, and error bars indicate standard deviations over this set. The temperature is the same as in Fig. 1. Dashed lines between points are drawn to guide the eye.

The sharp structural transition around *κ*_B_ ≈ κ^*^ is reminiscent of experiments showing that very few mutational steps (or even a single step) is sufficient to tip the balance from G_A_ to G_B_, or vice versa, for carefully selected mutational pathways^15^. Moreover, the minimum in the total folded population *P*_tot_ = *P*_A_ + *P*_B_ at *κ*_B_ ≈ κ^*^ (see Fig. 2a) is in line with the partial loss of stability seen for G_A_ and G_B_ sequences close to the transition point, e.g., G_A_98 and G_B_98^15^, as well as for other fold switching proteins^1,27^. These results allow us to interpret *κ*_B_ as a continuous parameter mimicking the number of steps taken along a mutational pathway connecting the G_A_ and G_B_ folds. The point *κ*_B_ = κ^*^ thus represents a sequence located on the border between G_A_ and G_B_. Although a sequence with a perfect G_A_ and G_B_ population balance was not reported, it has been found for other fold switching systems, e.g., the E48S variant of RfaH^7^ and the N11L mutant of the Switch Arc protein^28^. At *κ*_B_ = κ^*^, *P*_tot_ ≈ 0.82 meaning there is a minor population of U under these conditions. It is possible to achieve a higher *P*_tot_ while maintaining the G_A_ and G_B_ population balance by lowering the temperature below *T*_0_ and adjusting κ^*^ (see Supplementary Fig. S1). In the following, we focus our analysis on *T*_0_ and refer to our *κ*_B_ = κ^*^ model protein as ${{{{{{{{\rm{G}}}}}}}}}_{{{{{{{{\rm{AB}}}}}}}}}^{* }$.

### Macromolecular crowding effects on the G_A_/G_B_ fold switch

Next we introduce spherical crowder particles with an effective radius *R*_c_ = 12.5 Å (see “Methods”) into our simulation box, thereby probing the effect of volume exclusion on the G_A_/G_B_ switch from objects of roughly the size of the protein chain in either folded state. Because of steric repulsions, the protein chain must at all times avoid the space occupied by the crowders. Such loss of free volume typically stabilizes the native state (N) of single-fold proteins because any extended conformation in U becomes entropically disfavored relative to compact, folded conformations^29^. The same argument can be applied to each fold of a metamorphic protein. Hence, the overall stability of all folded states should increase. Indeed, as shown in Fig. 2a, the addition of crowders increases the total population *P*_tot_ = *P*_A_ + *P*_B_ across all values of *κ*_B_. Interestingly, poor stability is a common feature of fold-switching proteins^1^. For example, sequences on either side of the G_A_/G_B_ switch point exhibit reduced stabilities relative to wild-type G_A_ or G_B_^15^. Crowding effects, if indeed providing an overall stabilization, might therefore alleviate the partial loss of stability suffered by bridge sequences in evolutionary fold-switch transitions^30^.

To investigate how the relative population of the G_A_ and G_B_ folds is affected by crowders we focus on ${{{{{{{{\rm{G}}}}}}}}}_{{{{{{{{\rm{AB}}}}}}}}}^{* }$. Figure 2b shows that, as *ϕ*_c_ increases, the population balance exhibited by ${{{{{{{{\rm{G}}}}}}}}}_{{{{{{{{\rm{AB}}}}}}}}}^{* }$ at *ϕ*_*c*_ = 0 swings toward G_B_ at the expense of G_A_, i.e., *P*_B_ increases while *P*_A_ decreases. The effect on ${{{{{{{{\rm{G}}}}}}}}}_{{{{{{{{\rm{AB}}}}}}}}}^{* }$ is not small. For example, *P*_A_/*P*_B_ ≈ 4 at *ϕ*_c_ = 0.44 as compared to ≈ 1 at *ϕ*_c_ = 0. Hence, the effect of steric repulsions between crowders and protein is to favor to G_B_ over G_A_.

To quantitatively analyze this population shift we apply scaled particle theory (SPT)^31^. In this theory, the free energy cost of inserting a hard sphere of radius *R* into a fluid of hard spheres of radii *R*_c_ with packing fraction *ϕ*_c_ can be analytically expressed (see “Methods”). SPT has been used to model crowding-induced changes to the unfolding free energy Δ*F*_unf_ = *F*_U_ − *F*_N_, where *F*_U_ and *F*_N_ are the free energies of the U and N states, respectively^29,32,33^. Here we adapt SPT to fold switching by treating the G_A_ and G_B_ folds as spheres of radii *R*_A_ and *R*_B_. With the parametrization *R*_A_ = *R*_0_ − *δ* and *R*_B_ = *R*_0_ + *δ*, where *R*_0_ and *δ* are two parameters, the free energy difference can be written:

1 $$\beta \Delta {F}_{{{{{{{{\rm{SPT}}}}}}}}}=6\left[\left(a+2ab+a{b}^{2}+\frac{{a}^{3}}{3}\right)\psi +(3ab+3a{b}^{2}+{a}^{3}){\psi }^{2}+(3a{b}^{2}+{a}^{3}){\psi }^{3}\right],$$

where *a* = *δ*/*R*_c_, *b* = *R*_0_/*R*_c_, *ψ* = *ϕ*_c_/(1 − *ϕ*_c_) and *β* = 1/*k*_B_*T*. We fit the measured crowding-induced changes in free energy of fold switching, $\Delta {F}_{{{{{{{{\rm{switch}}}}}}}}}={F}_{{{{{{{{\rm{B}}}}}}}}}-{F}_{{{{{{{{\rm{A}}}}}}}}}=-{k}_{{{{{{{{\rm{B}}}}}}}}}T\ln [{P}_{{{{{{{{\rm{B}}}}}}}}}/{P}_{{{{{{{{\rm{A}}}}}}}}}]$ to Eq. (1) with *δ* as a single free parameter, fixing ${R}_{{{{{{{{\rm{0}}}}}}}}}={R}_{{{{{{{{\rm{g}}}}}}}}}^{{{{{{{{\rm{av}}}}}}}}}+{\sigma }_{{{{{{{{\rm{b}}}}}}}}}$, where ${R}_{{{{{{{{\rm{g}}}}}}}}}^{{{{{{{{\rm{av}}}}}}}}}=10.9$ Å is the average radius of gyration of the G_A_95 and G_B_95 native structures (see Fig. 1a), and *σ*_b_ = 4.0 Å is the radius of the beads in our protein chain. The fit is shown in Fig. 2c and gives *δ* = − 0.35 ± 0.03 Å. The size difference 2*δ* is in rough agreement with that calculated for the radii of gyration of the G_A_95 and G_B_95 native structures, ${R}_{{{{{{{{\rm{g}}}}}}}}}^{{{{{{{{\rm{A}}}}}}}}}=11.4$ Å and ${R}_{{{{{{{{\rm{g}}}}}}}}}^{{{{{{{{\rm{B}}}}}}}}}=10.5$ Å. The quality of the fit (*χ*^2^/(*n* − 1) = 22, sample size *n* = 7) indicates, however, that SPT does not fully describe the observed crowding effects on Δ*F*_switch_. A better agreement can be obtained by applying the generalized fundamental measure theory (GFMT) of Qin and Zhou^34,35^, which takes into account both the shape of protein conformations and their fluctuations (see Fig. 2c). The generally good agreement obtained for GFMT indicates, in particular, that accounting for fluctuations in chain size, which are absent in SPT, is necessary to describe the observed *ϕ*_c_-dependence of Δ*F*_switch_.

### Disordered tails control the crowding effect on the fold switch

The two terminal segments of the G_A_95 structure, residues 1-7 and 53-56, are intrinsically disordered (see Fig. 1a). Hence, the G_A_-to-G_B_ fold switch involves a disorder-order transition of these tail regions. Given their flexible nature, it is likely that the tails contribute substantially to the volume excluded by the protein when occupying the G_A_ fold. Indeed, if the terminal segments are ignored, the radius of gyration of G_A_95 is reduced by ≈22%, ${R}_{{{{{{{{\rm{g}}}}}}}}}^{{{{{{{{\rm{A}}}}}}}},8-52}=8.9$ Å. By contrast, the radius of gyration of G_B_95 determined over the same segment is ${R}_{{{{{{{{\rm{g}}}}}}}}}^{{{{{{{{\rm{B}}}}}}}},8-52}=10.8$ Å, which is a slight increase compared to value for the full chain. Together with the poor fit with SPT (see Fig. 2c), these results suggest a potential role for the tail segments in how the G_A_/G_B_ fold switch is impacted by crowding.

**Fig. 3:**
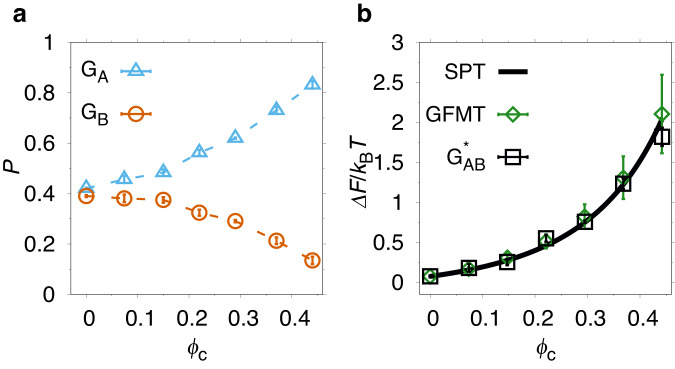
Disordered tail segments control crowding effects on the G_A_/G_B_ fold switch. Results are shown for simulations with a modified potential energy function that ignores steric repulsions between any crowder and beads in chain segments 1-7 and 53-56 (see text). **a** G_A_ (triangles) and G_B_ (circles) fold populations *P* as functions of *ϕ*_c_. **b** Fit of Δ*F*_switch_ (squares) to scaled particle theory (solid curve). Green rhombuses are Δ*F*_switch_ values calculated as in Fig. 2 but with GFMT applied only to the chain segment 8-52. The temperature is the same as in Fig. 1.

To show that this is indeed the case, we carry out crowding simulations with a modified potential energy function, ${E}_{{{{{{{{\rm{mod}}}}}}}}}^{({{{{{{{\rm{db}}}}}}}})}$, in which all crowder-protein interactions have been turned off for residues in the 1-7 and 53-56 regions. Hence, in these simulations, the N- and C-terminal segments become invisible to the crowders, which thus freely overlap with the residues. Although unphysical, this computational experiment logically tests the role of the tail regions in our model under crowded conditions. Note that crowders can overlap with the tails regardless of which state is populated by the protein. Moreover, at *ϕ*_c_ = 0, the model remains the same because intra-chain interactions are unaffected. The results are shown in Fig. 3a. Strikingly, with the modified potential ${E}_{{{{{{{{\rm{mod}}}}}}}}}^{({{{{{{{\rm{db}}}}}}}})}$, the impact of crowding reverses such that the G_A_ fold becomes increasingly favored over G_B_ with increasing *ϕ*_c_. Moreover, the fit to SPT (see Fig. 3b), obtained using *R*_0_ = 13.9 Å and giving *δ* = 0.42 ± 0.01 Å, is now much better (*χ*^2^/(*n* − 1) ≈ 1.5). We also carried out simulations with the modifications in ${E}_{{{{{{{{\rm{mod}}}}}}}}}^{({{{{{{{\rm{db}}}}}}}})}$ applied separately to the 1-7 and 53-56 regions. As it turns out, the effects on Δ*F*_switch_ is roughly additive (see Supplementary Fig. S2), suggesting that the two tail regions independently reduce the volume available to the crowders. Taken together, our computational experiment shows that the volume excluded by the disordered tails in the G_A_ fold is the dominant factor affecting the balance between the folds in the presence of crowders.

### Comparing with crowding effects on single-fold proteins

**Fig. 4:**
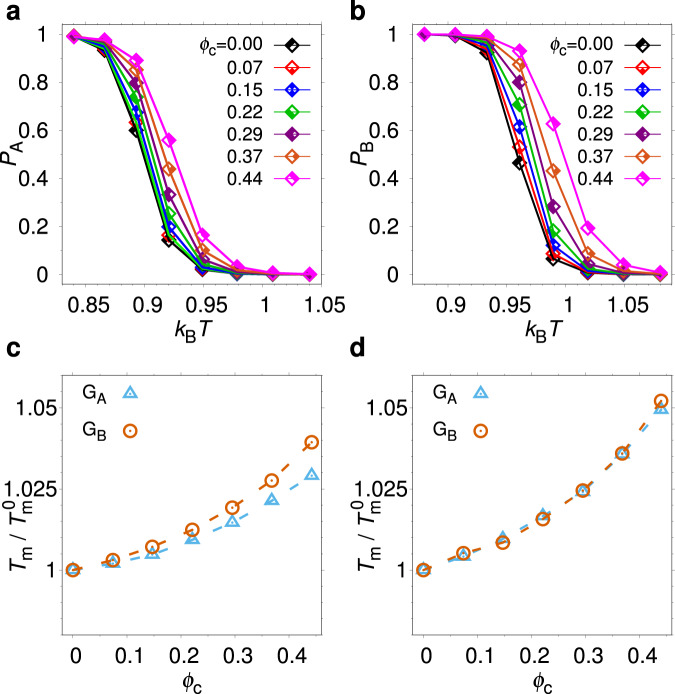
Crowding effects on single-fold G_A_ and G_B_ proteins. **a** G_A_ fold population, *P*_A_, obtained with our dual-basin structure-based model with weak G_B_ contacts (*κ*_B_ = 0.85), as function of temperature. **b** G_B_ fold population, *P*_B_, obtained with the same model but with strong G_B_ contacts (*κ*_B_ = 1.00). **c** Midpoint temperature, *T*_m_, as function of *ϕ*_c_. *T*_m_ is obtained by fitting the folding curves in (**a**) and (**b**) to a two-state model. **d** *T*_m_ as function of *ϕ*_c_, obtained with single-basin structure-based models for G_A_ and G_B_. In both (**c**) and (**d**), ${T}_{{{{{{{{\rm{m}}}}}}}}}^{0}$ is the value of *T*_m_ at *ϕ*_c_ = 0.

Above we have shown that the crowders induce a population shift in ${{{{{{{{\rm{G}}}}}}}}}_{{{{{{{{\rm{AB}}}}}}}}}^{* }$, which is due to the presence of disordered tails. For single-fold (monomorphic) proteins, purely repulsive crowders typically enhance the stability of N^36^. Naively, one may therefore expect that N of monomorphic G_B_ (*κ*_B_ > κ^*^) would be more strongly stabilized by the crowders than monomorphic G_A_ (*κ*_B_ < κ^*^). To test this idea, we determine the folding midpoint temperature, *T*_m_, for the model proteins with *κ*_B_ = 0.85, which adopts the single fold G_A_, and *κ*_B_ = 1.00, which adopts the single fold G_B_ (see Fig. 1), over a range of *ϕ*_c_. As seen in Fig. 4a–c, both proteins exhibit a monotonic increase in *T*_m_ with increasing *ϕ*_c_, indicating stabilization. The relative increase in *T*_m_ for monomorphic G_B_ is indeed somewhat larger than for monomorphic G_A_. The difference is relatively small, however. We also perform similar simulations using the single-basin energy functions *E*^(A)^ and *E*^(B)^, (see “Methods”) which lack entirely a bias toward the alternative fold. For these models, the crowding-induced increases in *T*_m_ are almost identical (see Fig. 4d). Taken together, these results suggest that determining the crowding response of a fold switcher with two “co-existing” folds may not be easily obtained from experiments on single-fold proteins representative of the two folds.

### The unfolded state changes character across the fold switch

The results in Fig. 4 are at first surprising because Δ*F*_switch_ for a fold switching protein can be obtained from the relation:

2 $$\Delta {F}_{{{{{{{{\rm{switch}}}}}}}}}=\Delta {F}_{{{{{{{{\rm{unf}}}}}}}}}^{{{{{{{{\rm{A}}}}}}}}}-\Delta {F}_{{{{{{{{\rm{unf}}}}}}}}}^{{{{{{{{\rm{B}}}}}}}}},$$

where $\Delta {F}_{{{{{{{{\rm{unf}}}}}}}}}^{{{{{{{{\rm{A}}}}}}}}}={F}_{{{{{{{{\rm{U}}}}}}}}}-{F}_{{{{{{{{\rm{A}}}}}}}}}$ and $\Delta {F}_{{{{{{{{\rm{unf}}}}}}}}}^{{{{{{{{\rm{B}}}}}}}}}={F}_{{{{{{{{\rm{U}}}}}}}}}-{F}_{{{{{{{{\rm{B}}}}}}}}}$ are defined in direct analogy with the unfolding free energy of a single fold protein. Equation (2) expresses that a decrease in Δ*F*_switch_ results when the crowding-induced stabilization of fold G_B_ relative to U is stronger than the stabilization of fold G_A_. However, Eq. (2) is only guaranteed to hold when Δ*F*_switch_, $\Delta {F}_{{{{{{{{\rm{unf}}}}}}}}}^{{{{{{{{\rm{A}}}}}}}}}$ and $\Delta {F}_{{{{{{{{\rm{unf}}}}}}}}}^{{{{{{{{\rm{B}}}}}}}}}$ are determined for the same protein for which U provides a common reference. We therefore examine if the drastic structural shift for low-energy (folded) conformations in the G_A_/G_B_ fold switch is accompanied by changes in U.

**Fig. 5:**
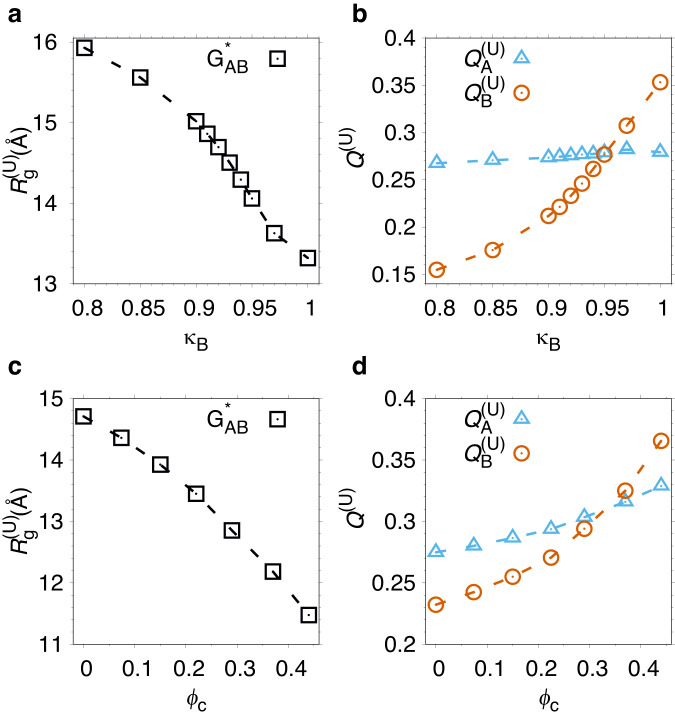
Changes to the unfolded state character across the fold switch. **a** ${R}_{{{{{{{{\rm{g}}}}}}}}}^{({{{{{{{\rm{U}}}}}}}})}$, **b** ${Q}_{{{{{{{{\rm{A}}}}}}}}}^{({{{{{{{\rm{U}}}}}}}})}$ (triangles) and ${Q}_{{{{{{{{\rm{B}}}}}}}}}^{({{{{{{{\rm{U}}}}}}}})}$ (circles) as functions of the contact strength *κ*_B_ at *ϕ*_c_ = 0, where ${R}_{{{{{{{{\rm{g}}}}}}}}}^{({{{{{{{\rm{U}}}}}}}})}$, ${Q}_{{{{{{{{\rm{A}}}}}}}}}^{({{{{{{{\rm{U}}}}}}}})}$, and ${Q}_{{{{{{{{\rm{B}}}}}}}}}^{({{{{{{{\rm{U}}}}}}}})}$ are the radius of gyration, fraction of G_A_ contacts, and fraction of G_B_ contacts, respectively, determined for the unfolded state, U. **c** ${R}_{{{{{{{{\rm{g}}}}}}}}}^{({{{{{{{\rm{U}}}}}}}})}$, **d** ${Q}_{{{{{{{{\rm{A}}}}}}}}}^{({{{{{{{\rm{U}}}}}}}})}$ (triangles) and ${Q}_{{{{{{{{\rm{B}}}}}}}}}^{({{{{{{{\rm{U}}}}}}}})}$ (circles) as functions of *ϕ*_c_, obtained for ${{{{{{{{\rm{G}}}}}}}}}_{{{{{{{{\rm{AB}}}}}}}}}^{* }$ (*κ*_B_ = 0.92). The temperature is the same as in Fig. 1.

We first characterize U across the fold switch in the absence of crowders, i.e., upon changing the contact strength *κ*_B_, as shown in Fig. 5a, b. With increasing *κ*_B_, and therefore increasing G_B_ population, the unfolded state radius of gyration ${R}_{{{{{{{{\rm{g}}}}}}}}}^{({{{{{{{\rm{U}}}}}}}})}$ decreases. Additionally, U becomes more “G_B_-like” as shown by the increase in ${Q}_{{{{{{{{\rm{B}}}}}}}}}^{({{{{{{{\rm{U}}}}}}}})}$, i.e., the fraction of formed G_B_ contacts in U. These results are in line with simulations of single-fold proteins showing that native contacts in *β*-proteins tend to promote chain collapse during folding more efficiently than *α*-proteins^37^. In the crowding-induced G_A_-to-G_B_ fold switch we similarly find a compaction of U (see Fig. 5c, d). For *ϕ*_c_ > 0.20, ${R}_{{{{{{{{\rm{g}}}}}}}}}^{({{{{{{{\rm{U}}}}}}}})}$ becomes smaller than for any value of *κ*_B_ in the case of no crowders. Moreover, ${Q}_{{{{{{{{\rm{A}}}}}}}}}^{({{{{{{{\rm{U}}}}}}}})}$ and ${Q}_{{{{{{{{\rm{B}}}}}}}}}^{({{{{{{{\rm{U}}}}}}}})}$ both increase with *ϕ*_c_. In summary, fold switching driven either by mutation or crowding substantially impacts the structural characteristics of U. Both chain compaction and the formation of residual structure due to crowding have been observed for various single-fold proteins^38,39^.

## Discussion

Fold switching in proteins involves major structural changes, including in shape and amino acid composition of surface regions. As a result, fold switching should be inherently susceptible to crowding effects. Here we tested this idea by applying a dual-basin structure-based protein model and purely repulsive crowders to the G_A_/G_B_ fold switch. We found that the addition of crowders indeed alters the free energy balance between the two folds. The effect increases monotonically with *ϕ*_c_. At *ϕ*_c_ = 0.44, the change in Δ*F*_switch_ is ≈ 2*k*_B_*T* in magnitude. While no experiment probing crowding effects on the G_A_/G_B_ fold switch is available for comparison, a key role for molecular shape in crowding has been demonstrated in a study that exploited alternative dimer forms of two almost identical sequences^40^. Very recently, it was shown using nuclear magnetic resonance spectroscopy that the addition of 90 g/l Ficoll, polyethylene glycol or BSA to the solution impacted the relative fold population of the two metamorphic proteins KaiB and XCL1^41^.

Our results show that crowding effects on the G_A_/G_B_ system may be determined by chain segments at the N- and C-terminal ends, which are intrinsically disordered only in the G_A_ fold. The volume excluded by these disordered segments leads to an entropic stabilization of G_B_ relative to G_A_. Interestingly, order-disorder transitions occur frequently in protein fold switching^1^. One example besides G_A_/G_B_ is human chemokine XCL1, which switches folds upon dimerization. In its monomeric (chemokine) fold, XCL1 adopts an *α*-helix in its C-terminal region, which becomes disordered when the protein transforms to its dimeric fold-switched state^12^. It should be pointed out that crowder interactions other than hard-core steric repulsions can modify the crowding effects. For single-fold proteins, nonspecific attractive (soft) interactions between protein and crowders generally counteract the stabilizing effect of volume exclusion^42^, and can even lead to a net destabilization^43^.

Most studies on fold switching have quite naturally focused on the structure and dynamics of the different folded states, and their interconversions. However, our simulations of the G_A_/G_B_ switch reveal that fold switching may be accompanied by substantial changes in U (see Fig. 5). Under conditions favoring G_A_, we find that U is rather expanded and dominated by local contacts while becoming more compact and forming more non-local contacts as the conditions shift to favor G_B_. In previous simulations of the metamorphic protein RfaH^25^, we showed that the isolated C-terminal domain, which adopts a stable *β*-barrel in isolation, exhibits a propensity for *α*-helical structure in U. This helical propensity was demonstrated experimentally by Zuber et al.^27^, who suggested further that the presence of residual helical structure may help initiate the reverse fold switching of RfaH, i.e., the transformation from the *β*-barrel to its alternative all-*α* fold. Taken together, the above considerations suggest that an improved understanding of the unfolded state of metamorphic proteins may give further insights into fold switching mechanisms, as well as effects from crowding.

In addition to changes to the relative population of the two folds, we have found that the presence of crowders increases the total population of the G_A_ and G_B_ folds relative to U. An overall stabilization of ordered states might be especially beneficial to fold-switching proteins, which often exhibit reduced stabilities^1^. Poor stabilities of bridge sequences at the border between folds may hamper evolutionary transitions^16,44,45^. A recent study suggests fold switching within the context of multidomain proteins, in which non-switching domains can act as stabilizing scaffolds, may help stabilize such bridge sequences and facilitate fold transitions^13^. Our results suggest that additional stabilization may be provided by crowding effects.

Our study opens up for additional experimental and theoretical investigations into the effects of crowding on fold switching. Recent advances in the fold switching field are improving our understanding of this phenomenon within functional^27,46^ and evolutionary^3,12,13^ contexts. These efforts should also include a characterization of the impact of crowding effects on equilibrium and kinetic properties of fold switching proteins.

## Model and methods

### Native structures and contact maps

The experimentally determined structures of G_A_95 (PDB id 2KDL) and G_B_95 (2KDM)^15^ were downloaded from the Protein Data Bank. Contact maps for 2KDL and 2KDM were obtained as prescribed by the shadow map method^47^ and contained 106 and 145 contacts, respectively.

### Observables

The fractions of native contacts were determined using *Q*_A_ = *N*_A_/106 and *Q*_B_ = *N*_B_/145, where *N*_A_ (*N*_B_) is the number of G_A_ (G_B_) contacts formed. A contact between two amino acids i and j was considered formed if ${r}_{{{{{{{{\rm{ij}}}}}}}}} < 1.2{r}_{{{{{{{{\rm{ij}}}}}}}}}^{0}$, where *r*_ij_ is the distance between the C_*α*_ atoms and ${r}_{{{{{{{{\rm{ij}}}}}}}}}^{0}$ is the distance in the native structure (2KDL or 2KDM). In determining the fold populations, *P*_A_ and *P*_B_, we classified a conformation to be in the G_A_ fold if ${N}_{{{{{{{{\rm{A}}}}}}}}} > {N}_{{{{{{{{\rm{A}}}}}}}}}^{{{{{{{{\rm{cut}}}}}}}}}=58$ and in the G_B_ fold if ${N}_{{{{{{{{\rm{B}}}}}}}}} > {N}_{{{{{{{{\rm{B}}}}}}}}}^{{{{{{{{\rm{cut}}}}}}}}}=76$, where ${N}_{{{{{{{{\rm{A}}}}}}}}}^{{{{{{{{\rm{cut}}}}}}}}}$ and ${N}_{{{{{{{{\rm{B}}}}}}}}}^{{{{{{{{\rm{cut}}}}}}}}}$ were determined based on the free energy profiles *F*(*N*_A_) and *F*(*N*_B_) for ${{{{{{{{\rm{G}}}}}}}}}_{{{{{{{{\rm{AB}}}}}}}}}^{* }$ (see Supplementary Fig. S3).

### Coarse-grained model for protein fold switching

Simulations were carried out using a dual-basin structure-based model in which each amino acid is represented by a single bead located on the C_*α*_-atom position. The starting point for developing this model was a modified version of the single-basin structure-based model developed previously^18^. The single-basin model has a potential energy function with 5 terms, *E* = *E*_bond_ + *E*_bend_ + *E*_torsion_ + *E*_rep_ + *E*_cont_, representing bond stretching, bond flexing, torsional rotations, repulsions between bead pairs, and attractive native contact interactions. We applied this model separately to the native structures of G_A_95 and G_B_95 resulting in two structure-based energy functions, *E*^(A)^ and *E*^(B)^, with single basins of attraction (either the G_A_ fold or the G_B_ folds). Using the exponentially-weighted mixing approach of Best et al.^48^, we then merged *E*^(A)^ and *E*^(B)^ into a single (dual basin) energy function, *E*^(db)^. The strength of G_A_ and G_B_ contacts, *κ*_A_ and *κ*_B_, were left as free parameters in *E*^(db)^, allowing the relative depth of the G_A_ and G_B_ basins of attraction to be controlled. Full details of the model are given in the Supporting Information (see Supplementary Notes).

### Excluded volume crowders

Crowder-crowder and crowder-protein interactions are described using the potential function^29^:

3 $$V(r)=\epsilon {\left(\frac{\sigma }{r-\rho +\sigma }\right)}^{12}$$

for distances *r* > *ρ* − *σ*, and *V*(*r*) = *∞* otherwise. Hence, our crowders have a soft repulsive shell over a hard core. The parameters *ρ* and *σ* control the range of the interaction and the width of the soft repulsive shell, respectively. We determined these parameters using *σ* = *σ*_i_ + *σ*_j_ and *ρ* = *R*_i_ + *R*_j_, where i and j are two interacting elements. When i, j are crowders we set *σ*_i_ = *σ*_j_ = 3 Å and *R*_i_ = *R*_j_ = 12 Å, and when one of i, j is a crowder and the other is a chain bead we set for the bead (assuming j) *σ*_j_ = *R*_j_ = *σ*_b_, where *σ*_b_ = 4 Å is the bead radius. With this choice of *ρ* and *σ*, an approximate value for the crowder radius *R*_c_ is ≈ 12 Å. A more precise value was obtained from the radial distribution function *g*(*r*) for a large crowder-only system, which indicated 12.5 Å (see Supplementary Fig. S4). We therefore use *R*_c_ = 12.5 Å for the crowder radius throughout this work. The crowder concentration, as quantified by the fraction of the total simulation volume *V* occupied by the crowders, is then given by ${\phi }_{{{{{{{{\rm{c}}}}}}}}}=4\pi {R}_{{{{{{{{\rm{c}}}}}}}}}^{3}{N}_{{{{{{{{\rm{c}}}}}}}}}/3V$. In our simulations, the number of crowder particles *N*_c_ ranges from 9 for *ϕ*_c_ = 0.073 to 54 for *ϕ*_c_ = 0.442.

### Langevin dynamics

Conformational sampling was carried out using Langevin dynamics following our previous approach^18^. The time evolution of the system is then governed by the equation, $m\dot{v}(t)={F}_{{{{{{{{\rm{conf}}}}}}}}}-m\gamma v(t)+\eta (t)$, where *m*, *v*, $\dot{v}$, *γ*, *F*_conf_ and *η*(*t*) are the mass, velocity, acceleration, friction coefficient, conformational force and random force, respectively. The random force *η*(*t*) is drawn from a Gaussian distribution, the variance of which sets the temperature of the system. For computational reasons, simulations were carried out in the low-friction (underdamped) limit, where −*mγv*(*t*) is small relative to the inertial term $m\dot{v}(t)$. In this limit, a natural unit of time for the dynamics is $\tau =\sqrt{m{l}^{2}/\epsilon }$^49^, where *ϵ* is the magnitude of typical interactions and *l* is a length scale, which we set to 4 Å. The friction coefficient for beads was taken to be *γ*_b_ = 0.05*τ*^−1^. Units were set so that the mass of a bead is *m*_b_ = 1.0. Numerical integration of the equation of motion was carried out using the velocity form of the Verlet algorithm^50^ with an integration time step *δt* = 0.005*τ*. For crowders, the mass and friction coefficient were set to *m*_c_ = 9.0 and *γ*_c_ = 0.017*τ*^−1^.

### Simulation and analysis details

Simulations were carried out by placing the protein and crowders in a cubic box with side 100 Å. Periodic boundary conditions were applied. Langevin dynamics simulations were used to determine the equilibrium behavior of various systems characterized by different G_B_ contact strengths *κ*_B_ and crowder concentrations *ϕ*_c_. Simulations were performed at either fixed temperature or using simulated tempering^51^, in which temperature changes dynamically between a predetermined set of values. In the simulated tempering runs, temperatures were updated every 100 time steps. For each system, 5-10 independent runs of (4 − 5) × 10^9^ time steps each were carried out and used to estimate averages and statistical uncertainties. All simulations were initiated from a random protein conformation (random torsional angles *ϕ*_i_) and random crowder positions, followed by a Monte Carlo-based relaxation step in which all hard core steric clashes were removed.

### Theory

Simulation results were analyzed using scaled particle theory (SPT)^31^ and generalized fundamental measure theory (GFMT)^34,35^. According to SPT, the free energy cost of inserting a hard sphere of radius *R* in a hard sphere fluid of particles with radius *R*_c_ is^31^:

4 $$\beta F=(3x+3{x}^{2}+{x}^{3})\psi +\left(\frac{9{x}^{2}}{2}+3{x}^{3}\right){\psi }^{2}+3{x}^{3}{\psi }^{3}-\ln (1-{\phi }_{{{{{{{{\rm{c}}}}}}}}}),$$

where *β* = 1/*k*_B_*T*, *T* is the temperature, *k*_B_ is the Boltzmann constant, $x=\frac{R}{{R}_{{{{{{{{\rm{c}}}}}}}}}}$, $\psi =\frac{{\phi }_{{{{{{{{\rm{c}}}}}}}}}}{1-{\phi }_{{{{{{{{\rm{c}}}}}}}}}}$, and *ϕ*_c_ is fluid volume fraction. Minton showed that SPT predicts a strong stabilizing effect on the native states of single-fold proteins if U is modeled as an ideal Gaussian chain^33^. Here SPT was used to model the G_A_/G_B_ folds as spheres of different radii.

GFMT accounts for geometric features of the protein structure using three quantities, linear size *l*_p_, surface area *s*_p_, and volume *ν*_p_. These quantities are obtained by sampling the crowder-excluded surface, which is influenced by the crowder radius, chain bead radius, and the protein conformation. The free energy cost of inserting a protein conformation into a crowder fluid is then estimated using^34,35^:

5 $$\beta F=\beta {\Pi }_{{{{{{{{\rm{c}}}}}}}}}{\nu }_{{{{{{{{\rm{p}}}}}}}}}+\beta {\gamma }_{{{{{{{{\rm{c}}}}}}}}}{s}_{{{{{{{{\rm{p}}}}}}}}}+\beta {\kappa }_{{{{{{{{\rm{c}}}}}}}}}{l}_{{{{{{{{\rm{p}}}}}}}}}-\ln (1-{\phi }_{{{{{{{{\rm{c}}}}}}}}}),$$

where Π_c_, *γ*_c_ and *κ*_c_ are osmotic pressure, surface tension, and bending rigidity (curvature), respectively, of the crowder fluid. To calculate the change in fold switching free energy at different *ϕ*_c_ using GFMT, we set the radii of crowder and beads to 12.5 and 4.0 Å, respectively. GFMT calculations were performed on 200 random chain conformations for each of the G_A_ and G_B_ folds, taken from our simulations of the model protein ${{{{{{{{\rm{G}}}}}}}}}_{{{{{{{{\rm{AB}}}}}}}}}^{* }$ at temperature *T*_0_.

### Supplementary information

Peer Review File

Supplementary Information

## Data Availability

Data sharing not applicable to this article as no datasets were generated or analysed during the current study.

## Code availability

Simulations were carried out using in-house software written in C. The software will be made available for academic use upon request to the corresponding author.
