# Supplementary material for: Simulations of a protein fold switch reveal crowding-induced population shifts driven by disordered regions
Source: Commun Chem. 2023 Sep 9;6:191. doi: 10.1038/s42004-023-00995-2 (PMC10492864; doi:10.1038/s42004-023-00995-2)
Supplement: Supplementary file 2 — Supplementary Information [file 42004_2023_995_MOESM2_ESM.pdf]

Supplementary Information for

Simulations of a protein fold switch reveal crowding-induced population shifts driven by disordered regions

Saman Bazmi[†], Bahman Seifi[†] and Stefan Wallin[†,*]

[†]*Department of Physics and Physical Oceanography,*

Memorial University of Newfoundland,

St Johns, NL A1B 3X7, Canada.

Contents

1 Supplementary Figures **3**

2 Supplementary Notes **7**

2.1 Development of computational model for the G_A/G_B fold switch 7

3 Supplementary References **12**

1 Supplementary Figures

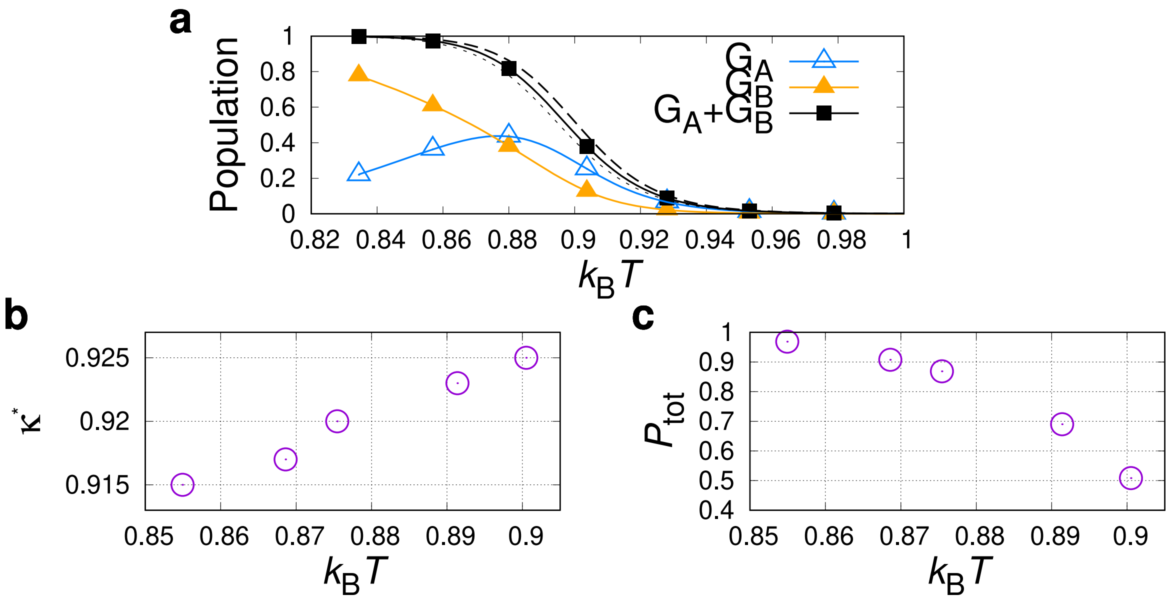

Figure S1: (a) G_A, G_B, and total G_A and G_B (P_{tot}) fold populations for the protein G^*_{AB}, i.e., $\kappa_B = 0.92$, as function of temperature T. Smooth curves are obtained from MBAR analysis [1]. (b) κ^* as function of T, obtained from simulations with $\kappa_B = 0.915, 0.917, 0.920, 0.923$ and 0.925. (c) P_{tot} as function of T, for the five κ_B values in (b). Shown in (a) is also P_{tot} for $\kappa_B = 0.915$ (short dashed curve) and $\kappa_B = 0.925$ (long dashed curve).

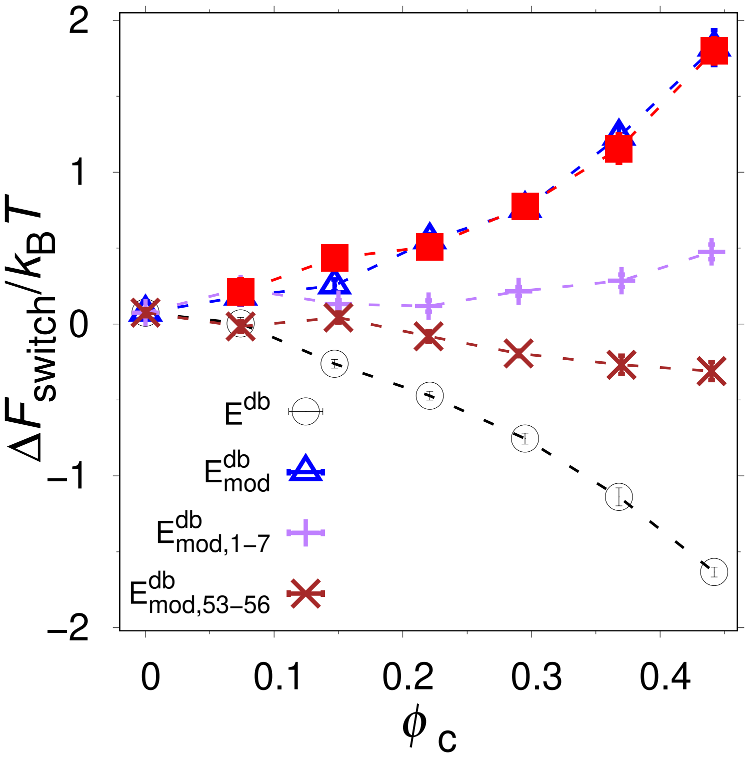

Figure S2: Crowder concentration (ϕ_c) dependence of the free energy of fold switching ($\Delta F_{\mathrm{switch}}$) for the protein $\mathrm{G}^*_{\mathrm{AB}}$ using four different potential energy functions: $E^{(\mathrm{db})}$ (original dual-basin model; see section 2.2 of this document), $E^{(\mathrm{db})}_{\mathrm{mod,1\text{-}7}}$ (crowder-bead interactions turned off for the segment 1-7), $E^{(\mathrm{db})}_{\mathrm{mod,53\text{-}56}}$ (crowder-bead interactions turned off for the segment 53-56), and $E^{(\mathrm{db})}_{\mathrm{mod}}$ (crowder-bead interactions turned off for both 1-7 and 53-56). Also shown, for $\phi_c > 0$, are the results obtained by adding the changes in $\Delta F_{\mathrm{switch}}$ for $E^{(\mathrm{db})}_{\mathrm{mod,1\text{-}7}}$ and $E^{(\mathrm{db})}_{\mathrm{mod,53\text{-}56}}$ relative to the original model $E^{(\mathrm{db})}$ (filled squares). The good agreement with the results for $E^{(\mathrm{db})}_{\mathrm{mod}}$ indicates that the effects of the modifications introduced in $E^{(\mathrm{db})}_{\mathrm{mod,1\text{-}7}}$ and $E^{(\mathrm{db})}_{\mathrm{mod,53\text{-}56}}$ are roughly additive in the quantity $\Delta F_{\mathrm{switch}}$.

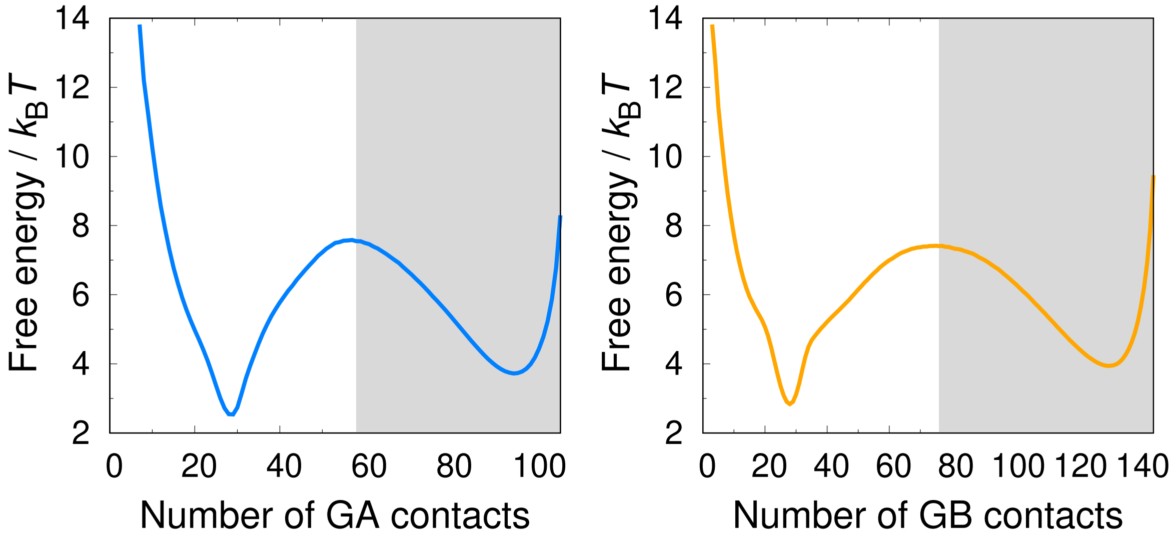

Figure S3: Free energy as function of the number of G_A contacts and the number of G_B contacts, obtained for G^*_{AB} at $k_B T_0 = 0.88$. In determining fold populations, we consider the G_A fold formed if the number of G_A contacts is > 58 and we consider the G_B fold formed if the number of G_B contacts is > 76, respectively (shaded areas).

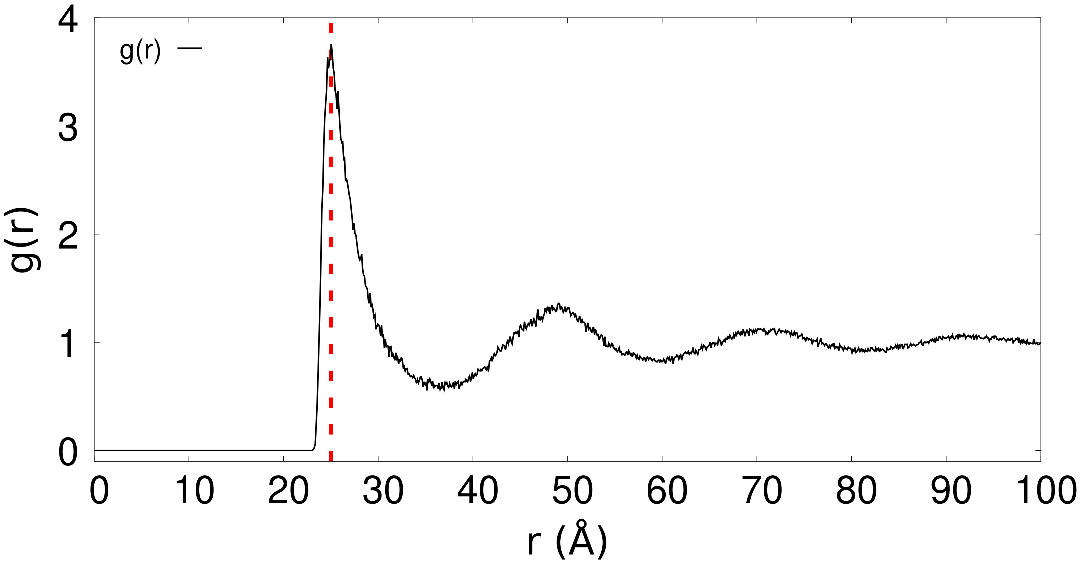

Figure S4: Pair correlation function, $g(r)$, obtained from a simulations of 1755 crowder particles in a cubic box with side 300 Å and periodic boundary conditions. The temperature is held fixed at T_0 ($k_B T_0 = 0.88$). An effective radius of the crowders ($R_c = 12.5$ Å) was determined based on the location of the first peak in $g(r)$, which occurs at the interparticle distance $r = 25$ Å (vertical dashed line).

2 Supplementary Notes

2.1 Development of computational model for the G_A/G_B fold switch

Single-basin structure-based model for protein folding

As a starting point for the development our dual-basin structure-based model, which we apply in this work to the G_A/G_B switch, we take the single-basin model for protein folding developed in Ref. [2]. We start by describing this single-basin model along with a modification introduced here to enhance the conformational specificity of native contact interactions. We find that the enhanced contact specificity is necessary to make the two folds structurally well defined in the dual-basin model. Geometrically, the protein is represented by beads located at the C_α atom positions. The conformation of an N-amino-acid chain can therefore be described by the bead positions $\mathbf{r}_i$, where $i = 1, ..., N$. Alternatively, a conformation can be described by the bond lengths, b_i, bond angles, θ_i, and dihedral angles, ϕ_i, defined by the $N-1$ (pseudo) C_α-C_α bonds of the chain. We denote by b_i^0, θ_i^0, and ϕ_i^0 the values of b_i, θ_i and ϕ_i in the native conformation. The potential energy E can be written as a sum of five terms:

$$\begin{aligned} E &= \sum_{i}^{\text{bonds}} K_b(b_i - b_i^0)^2 + \sum_{i}^{\text{angles}} K_\theta(\theta_i - \theta_i^0)^2 \\ &+ \sum_{i}^{\text{dihedrals}} K_\phi^{(1)}[1 - \cos(\phi_i - \phi_i^0)] + K_\phi^{(3)}[1 - \cos 3(\phi_i - \phi_i^0)]) \\ &+ \sum_{i<j-3}^{\text{nonnative}} \epsilon \left(\frac{\sigma}{r_{ij}}\right)^{12} + \sum_{i<j-3}^{\text{native}} \epsilon(h_{ij} - f_{ij}), \end{aligned} \tag{1}$$

where ϵ sets the energy scale of the model and $r_{ij} = |\mathbf{r}_j - \mathbf{r}_i|$. The first three terms represent bonded interactions with strengths set to $K_b = 100\epsilon$, $K_\theta = 20\epsilon$, $K_\phi^{(1)} = \epsilon$ and $K_\phi^{(3)} = 0.5\epsilon$. The fourth term represents steric repulsions between bead pairs that do not form a contact in the native structure. The repulsion range is set to $\sigma = 4$ Å. These first four terms in Eq. 1

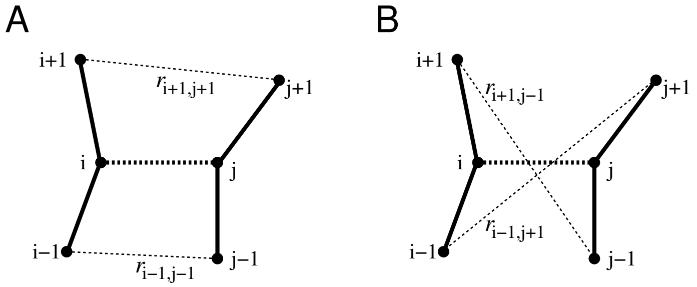

Figure N1: A contact between two non-terminal positions, i and j, (thick dashed line) has four different nearest neighbor-nearest neighbor distances (thin dashed lines): (A) $r_{\mathrm{i-1,j-1}}$ and $r_{\mathrm{i+1,j+1}}$ and (B) $r_{\mathrm{i-1,j+1}}$ and $r_{\mathrm{i+1,j-1}}$. In evaluating the factor $g_{\xi_2}(r'_{\mathrm{ij}})g_{\xi_2}(r''_{\mathrm{ij}})$ in Equation 1, r'_{ij} and r''_{ij} are the distances shown in (A), if $\Sigma_{\mathrm{A}} < \Sigma_{\mathrm{B}}$, or in (B), if $\Sigma_{\mathrm{B}} < \Sigma_{\mathrm{A}}$, where $\Sigma_{\mathrm{A}} = r^0_{\mathrm{i-1,j-1}} + r^0_{\mathrm{i+1,j+1}}$ and $\Sigma_{\mathrm{B}} = r^0_{\mathrm{i-1,j+1}} + r^0_{\mathrm{i-1,j+1}}$.

are identical to Ref. [2].

The final term in Eq. 1 represents native contact interactions, which in the previous model [2] were described by the Lennard-Jones potential $f_{\mathrm{LJ}}(r_{\mathrm{ij}}) = (r^0_{\mathrm{ij}}/r_{\mathrm{ij}})^{12} - 2(r^0_{\mathrm{ij}}/r_{\mathrm{ij}})^6$. Here we separate the interaction into a repulsive part (h_{ij}) and an attractive part (f_{ij}), such that they can be independently controlled. The repulsive part is described by a Weeks-Chandler-Anderson type function,

$$h_{\mathrm{ij}} = \begin{cases} \left(\frac{r^0_{\mathrm{ij}}}{r_{\mathrm{ij}}}\right)^{12} - 2\left(\frac{r^0_{\mathrm{ij}}}{r_{\mathrm{ij}}}\right)^{6} + 1, & \text{if } r_{\mathrm{ij}} < r^0_{\mathrm{ij}}\,, \\ 0, & \text{if } r_{\mathrm{ij}} \geq r^0_{\mathrm{ij}}\,, \end{cases} \tag{2}$$

where r^0_{ij} is the distance between beads i and j in the native structure. The attractive part takes the form

$$f_{\mathrm{ij}} = g_{\xi_1}(r_{\mathrm{ij}})g_{\xi_2}(r'_{\mathrm{ij}})g_{\xi_2}(r''_{\mathrm{ij}})\,, \tag{3}$$

where $g_\xi(r) = \exp[-(r - r^0)^2/2\xi^2]$. With the construct in Eq. 3, the distance r_{ij} as well as the two nearest neighbor distances, r'_{ij} and r''_{ij}, (see Figure N1) must assume their respective native values r^0_{ij}, r'^0_{ij} and r''^0_{ij} for ij to become a fully formed native contact, which then contributes $-\epsilon$ towards the total potential energy E. The parameter ξ_1 sets the width of the attractive well $-\epsilon g_{\xi_1}(r_{\mathrm{ij}})$. The combination of this attractive well and the repulsive part of the interaction results in a function, $h_{\mathrm{ij}} - g_{\xi_1}$, with gross features similar to a Lennard-Jones

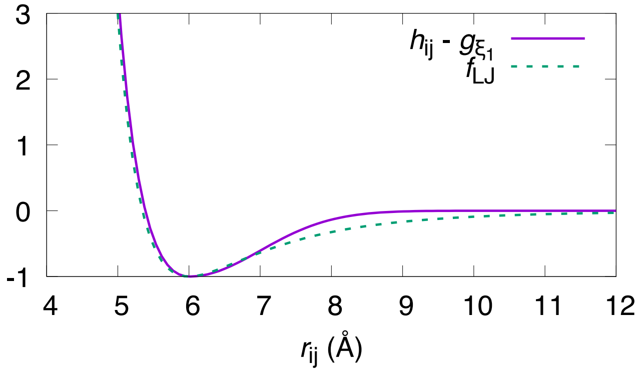

Figure N2: The potentials $h_{ij} - g_{\xi_1}$ and f_{LJ} (see text) as functions of r_{ij} using $r^0_{ij} = 6$ Å.

potential (see Figure N2).

The factor $g_{\xi_2}(r'_{ij})g_{\xi_2}(r''_{ij})$ is included in f_{ij} in order to increase the conformational specificity of the native interactions. For a contact between residues i and j, this factor promotes the local chain segments (i − 1, i, i + 1) and (j − 1, j, j + 1) to adopt relative orientations close to that found in the native structure. The strength of this effect is controlled by the parameter ξ_2. It is weak when $\xi_2 \gg \xi_1$ and becomes strong when $\xi_2 \approx \xi_1$. Test simulations on a few small single domain proteins show that decreasing ξ_2 leads to increased co-operativity in the folding transition (data not shown). We picked $\xi_1 = 1.0$ Å and $\xi_2 = 5.0$ Å. We note also that there are terms in Eq. 3 for which r'_{ij} or r''_{ij} is undefined because i or j is a terminal bead. In those cases, we set the corresponding factor g equal to unity.

The effect from the factor $g_{\xi_2}(r'_{ij})g_{\xi_2}(r''_{ij})$ in Eq. 3 is similar to so-called local-nonlocal coupling [3], which also leads to increased folding co-operativity. Our effect is not exactly the same, however, because it does not provide a direct constraint on the local internal conformation around beads i and j. Such a constraint does exist in local-nonlocal coupling.

Dual-basin structure-based model for fold switching

Next we extend the model of the previous section to a dual-basin (db) model, which provides bias towards two different reference structures "(a)" and "(b)". Such a bias can be achieved

by first obtaining the two single-basin energy potentials $E^{(\mathrm{a})}$ and $E^{(\mathrm{b})}$ using Eq. 1, and thereafter merging them into a single energy surface, $E^{(\mathrm{db})}$. Naively, one may attempt to put $E^{(\mathrm{db})} = E^{(\mathrm{a})} + E^{(\mathrm{b})}$. However, this strategy is problematic for some types of interactions, as pointed out by Ramirez-Sarmiento et al. [4]. For example, the sum of two quadratic bond terms $K_{\mathrm{b}}[(b_{\mathrm{i}} - b_{\mathrm{i}}^{(\mathrm{a})})^2 + (b_{\mathrm{i}} - b_{\mathrm{i}}^{(\mathrm{b})})^2]$ is another quadratic function with minimum at $(b_{\mathrm{i}}^{(\mathrm{a})} + b_{\mathrm{i}}^{(\mathrm{b})})/2$. Hence, this would abolish both minima. We combine the two single-basin potentials $E^{(\mathrm{a})}$ and $E^{(\mathrm{b})}$ using the procedure described below, which avoids these problems. This procedure is then applied to the $\mathrm{G_A}$ and $\mathrm{G_B}$ folds to produce the dual-basin potential used in this work.

Bonded terms. The bonded interactions are represented by the first three terms in Eq. 1. Consider two individual energy terms, $e^{(\mathrm{a})}(x)$ and $e^{(\mathrm{b})}(x)$, with global minimum at $x = x^{\mathrm{a}}$ and $x = x^{\mathrm{b}}$, respectively. The functions $e^{(\mathrm{a})}(x)$ and $e^{(\mathrm{b})}(x)$ could be, e.g., the bond angle terms corresponding to a particular bond, in which case $x = \theta_{\mathrm{i}}$. To "mix" $e^{(\mathrm{a})}(x)$ and $e^{(\mathrm{b})}(x)$ into a single function $e(x)$, we use [5]

$$e(x) = \beta_{\mathrm{mix}}^{-1} \ln \left[e^{-\beta_{\mathrm{mix}} e^{(\mathrm{a})}(x)} + e^{-\beta_{\mathrm{mix}} e^{(\mathrm{b})}(x)} \right] , \tag{4}$$

where β_{mix} is a parameter controlling the smoothness of the mixing. We pick $\beta_{\mathrm{mix}} = 10$ for the bond term, and $\beta_{\mathrm{mix}} = 5$ for the angle and torsion terms. Examples of three different terms for the $\mathrm{G_A}$ and $\mathrm{G_B}$ folds are given in Figure N3.

Non-bonded terms. For the native contact term, we include all contact interactions present in either $E^{(\mathrm{a})}$ or $E^{(\mathrm{b})}$. Although this is straightforward in principle, care must be taken to avoid double counting interactions for common contacts, i.e., contacts that occur in both (a) and (b). Moreover, we want to insert parameters κ_{A} and κ_{B} such that strengths of the attractive wells $-\epsilon f_{\mathrm{ij}}^{(\mathrm{a})}$ and $-\epsilon f_{\mathrm{ij}}^{(\mathrm{a})}$ can be controlled. Hence, our dual-basin contact

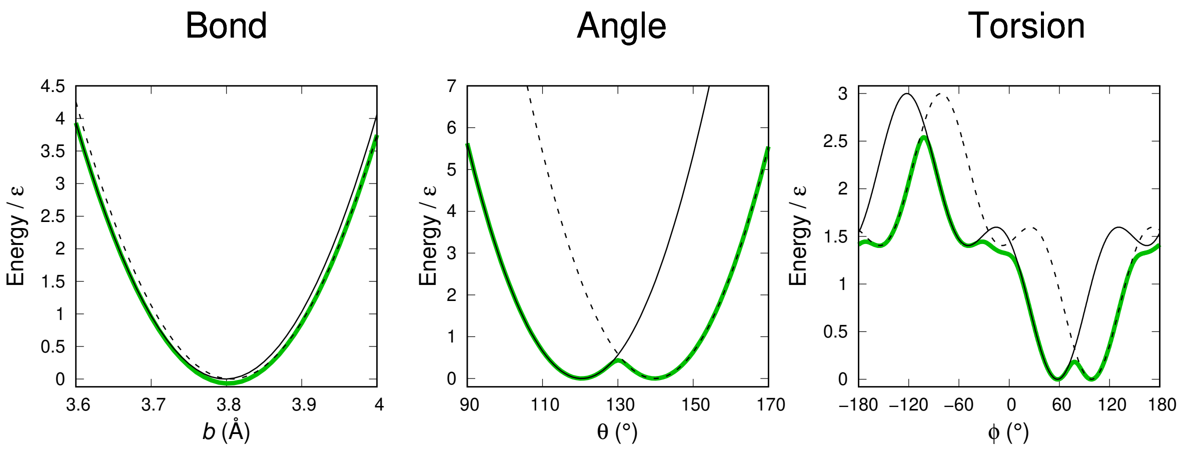

Figure N3: Examples of the merging of different bonded potentials for G_A and G_B (thin black solid/dashed curves) into a single potential (thick solid green curves) using the "mixing" equation 4 .

term becomes

$$\sum_{ij}^{(a)} \epsilon(h_{ij}^{(a)} - \kappa_A f_{ij}^{(a)}) + \sum_{ij}^{(b)} \epsilon(h_{ij}^{(b)} - \kappa_B f_{ij}^{(b)}) + \sum_{ij}^{\text{common}} \epsilon \left\{ \tilde{h}_{ij} - \max\left[\kappa_A f_{ij}^{(a)}, \kappa_B f_{ij}^{(b)}\right] \right\} ,$$

where the first two sums are taken over native contacts in (a) and native contacts in (b), respectively, *excluding* all common contacts, and the final sum is taken over these common contacts. Note that, for each common contact, only the energetically most favorable attraction is retained. The repulsive part, $\tilde{h}_{ij}$, is evaluated as h_{ij} using the smallest of the two reference distances, i.e., $r_{ij}^0 = \min\left[r_{ij}^{(a)}, r_{ij}^{(b)}\right]$. Picking r_{ij}^0 this way is necessary to guarantee that both conformations (a) and (b) can be formed without suffering a strong steric repulsion in one of the conformations, which would otherwise happen when $r_{ij}^{(a)}$ and $r_{ij}^{(b)}$ are very different. Note also that r_{ij}^0 for common contacts can be calculated before a simulation and that $\tilde{h}_{ij}$ does not change form during the simulation. The nonnative repulsive energy term, i.e., the fourth term in Eq. 1, is evaluated over all pairs ij that are not contacts in either (a) or (b).

3 Supplementary References

[1] M. R. Shirts and J. D. Chodera. Statistically optimal analysis of samples from multiple equilibrium states. *J Chem Phys*, 129:124105, 2008.

[2] S. Wallin and H. S. Chan. Conformational entropic barriers in topology-dependent protein folding: perspectives from a simple native-centric polymer model. *J Phys Condens Matter*, 18:S307, 2006.

[3] H. S. Chan, Z. Zhang, S. Wallin, and Z. Liu. Cooperativity, local-nonlocal coupling, and nonnative interactions: principles of protein folding from coarse-grained models. *Annu Rev Phys Chem*, 62:301–326, 2011.

[4] C. A. Ramirez-Sarmiento, J. K. Noel, S. L. Valenzuela, and I. Artsimovitch. Interdomain contacts control native state switching of RfaH on a dual-funneled landscape. *PLOS Comput Biol*, 11:e1004379, 2015.

[5] R. B. Best, Y. G. Chen, and G. Hummer. Slow protein conformational dynamics from multiple experimental structures: the helix/sheet transition of arc repressor. *Structure*, 13:1755–1763, 2005.
